# Supplementary material for: Anti-GD2 induced allodynia in rats can be reduced by pretreatment with DFMO
Source: PLoS One. 2020 Jul 22;15(7):e0236115. doi: 10.1371/journal.pone.0236115 (PMC7375533; doi:10.1371/journal.pone.0236115)
Supplement: S1 Table — Ion (m/z) transitions monitored, scheduled acquisition times (minutes), analyzer parameters (declustering (DP), entrance (EP) and collision cell (CXP) potentials) and analytical range (micromoles per liter; μL) for putrescine, difluoromethylornithine (DFMO), and stable-isotope labeled internal standards, 1,1,2,2,3,3,4,4-2H8-putrescine (d8-putrescine) and 5,5-2H2-ornitine (d2-ornithine). Numerals -1 indicate the transition used for quantification, and -2 the one used for confirmation. (DOCX) [file pone.0236115.s001.docx]

**S1 Table. Scheduled acquisition times of putrescine and DFMO**

| Compound | Parent (m/z) | Daughter (m/z) | time (min) | DP | EP | CE | CXP | min (µM) | max (µM) |
| --- | --- | --- | --- | --- | --- | --- | --- | --- | --- |
| putrescine-1 | 89.09 | 72.1 | 1.6 | 32 | 10 | 13 | 8 | 0.1 | 5 |
| putrescine-2 | 89.09 | 48.1 | 1.6 | 32 | 10 | 9 | 8 |  |  |
| DFMO-1 | 182.96 | 120.2 | 1.4 | 46 | 10 | 27 | 4 | 1 | 100 |
| DFMO-2 | 182.96 | 166.2 | 1.4 | 46 | 10 | 15 | 6 |  |  |
| d8-putrescine-1 | 97.14 | 80 | 1.6 | 32 | 10 | 13 | 8 |  |  |
| d2-ornithine | 135 | 72 | 1.4 | 36 | 10 | 22 | 8.1 |  |  |

**S1 Table.** Ion (m/z) transitions monitored, scheduled acquisition times (minutes), analyzer parameters (declustering (DP), entrance (EP) and collision cell (CXP) potentials) and analytical range (micromoles per liter; µL) for putrescine, difluoromethylornithine (DFMO), and stable-isotope labeled internal standards, 1,1,2,2,3,3,4,4-^2^H_8_-putrescine (d8-putrescine) and 5,5-^2^H_2_-ornitine (d2-ornithine). Numerals -1 indicate the transition used for quantification, and -2 the one used for confirmation.
