# Supplementary material for: Anti-GD2 induced allodynia in rats can be reduced by pretreatment with DFMO
Source: PLoS One. 2020 Jul 22;15(7):e0236115. doi: 10.1371/journal.pone.0236115 (PMC7375533; doi:10.1371/journal.pone.0236115)
Supplement: S1 Method — (DOCX) [file pone.0236115.s002.docx]

**S1 Method. Putrescene and DFMO measurement by HPLC and LC-MS/MS**

100µL of rat serum were vortex-mixed with 1mL of ice-cold methanol containing stable-isotope labeled internal standards (Cambridge Isotope Laboratories, Andover, MA), incubated for 30 minutes at -20°C and centrifuged at 17,136 x*g* at 5°C. Supernatants were evaporated to dryness in a centrifugal evaporator at 36°C (Savant SPD121P Speed Vac concentrator. Thermo Fisher, Asheville, NC, USA) and reconstituted in 50µL of mobile phase, by vortexing, orbital shaking and sonication, in sequence.

10µL aliquots were injected in the system using a Waters Acquity UPLC (Milford, MA), and isocratically separated at room temperature on a 150 x 2.1mm (5µm particle size) TSKgel Amide-80 column and guard cartridge containing carbamoyl (amide) covalently bonded silica at a flow rate of 0.3mL/min, delivered by the UPLC binary pump equipped with an in-line degasser. Mobile phase was 5% acetonitrile in 2mM ammonium formate + 0.1% formic acid (normal phase). Run time was 2.5min.

Compounds were assayed using a Sciex 4500 (Redwood City, CA) triple quadrupole mass spectrometer. The Turbo V source was operating in the positive electrospray ionization mode at 500°C and 5500 V. Nebulizer and heater gases were set at 40psi. Curtain gas was 30psi and Collision Gas (nitrogen) 9psi. Specific multiple reaction monitoring (MRM) transitions were optimized by infusion for putrescine (m/z 89.1→72.1), and 1,1,2,2,3,3,4,4-^2^H_8_-putrescine (d8-putrescine; m/z 97.1→80), using a collision energy (CE) of 13v, declustering potential (DP) of 32v at unit mass resolution. Difluoromethylornithine (DFMO) (m/z 182.96→120.2) and 5,5-^2^H_2_-ornitine (d2-ornithine; m/z 135→72) and scheduled acquisition times listed in S2 Table.

Samples are quantitated against at least 8 non-zero points calibration curve constructed by supplementing plasma with the appropriate amounts of putrescine, spermidine and DFMO.
