# Supplementary material for: Anti-GD2 induced allodynia in rats can be reduced by pretreatment with DFMO
Source: PLoS One. 2020 Jul 22;15(7):e0236115. doi: 10.1371/journal.pone.0236115 (PMC7375533; doi:10.1371/journal.pone.0236115)
Supplement: S2 Method — (DOCX) [file pone.0236115.s003.docx]

**S2 Methods. Putrescene, spermidine and spermine measurement by HPLC and LC-MS/MS using derivatives**

Polyamines were analyzed as the urethane derivatives of putrescine (di-isobutoxy-; ^i^BuO-), spermidine (tri- ^i^BuO-) and spermine (tetra- ^i^BuO-) and using stable-isotope dilution, ^2^H_8_-putrescine (di- ^i^BuO-d_8_-putrescine) and ^2^H_20_-spermine (tetra- ^i^BuO-d_20_-putrescine), as internal standards. Adapted from (22) and (23). Briefly, 25µL of serum were deproteinized with 4% trichloracetic acid. pH was adjusted to 9 in the supernatant by adding 50µL of 28% ammonium hydroxide and 50µL of 100mM ammonium formate buffer (pH 9), followed by reaction with 20µL of isobutyl chloroformate (Sigma Millipore, St. Louis, Missouri, USA) 15 minutes at 35°C. Carbamoyl derivatives were extracted by solid-phase extraction (SPE) on a 30mg Strata-X reversed phase cartridge (Phenomenex, Torrance, California, USA), by, sequentially, conditioning with 1mL of methanol, 1mL of water, loading the sample, washing with 5% acetonitrile, drying the cartridge at full vacuum and eluting them by adding 2 x 0.5mL of 90% acetonitrile +0.1% formic acid. Eluate was evaporated to dryness and reconstituted in 25µL of 50% acetonitrile+0.1% formic acid by vortexing, 15 minutes of orbital shaking, and 5 minutes sonication in a water bath, both at room temperature.

Liquid chromatography tandem mass spectrometry (LC-MS/MS) was conducted on a AP4000 MS (Foster City, California, USA) coupled to an Agilent 1200 series LC (Milford, Massachussets, USA) on a Kinetex C18 LC column (100x2.1mm; 5µm) running a 0.3mL/min gradient from 50% acetonitrile + 0.1% formic (B) to 100% B in 5 minutes, and re-equilibrating the column at 50% B after 1 minute at 100% B. 5µL were injected. Monitored transitions were: di-^i^BuO-putrescine: m/z 289.2>215 (CE 13v) (di-^i^BuO-d_8_-putrescine, m/z 297.2>223); tri-^i^BuO-spermidine: m/z 446.3>372 (CE 17v) and tetra- ^i^BuO-spermine: m/z 603.3>455 (CE 29v) (tetra- ^i^BuO-d20-spermine, m/z 623.3>475).

DFMO was assayed after direct extraction with ice-cold methanol (1:10; v/v), evaporation to dryness and reconstitution, same above steps, in 50µL of 5% acetonitrile in 3.6mM ammonium formate + 0.1% formic acid, that was the mobile phase used to analyze it in the same LC-MS/MS system, using a 150 x 2.1mm (5µm particle size) TSKgel Amide-80 column at a flow rate of 0.3mL/min. ^2^H_2_-ornithine (d_2_-ornithine) was used as internal standard. Monitored transitions were m/z 183>120.2 (CE 27v) for DFMO, and m/z 135>72 (CE 22v) for d_2_-ornithine.

Metabolite concentrations were calculated using the authentic standard in six to eight non-zero levels calibration curves within 85-115% (80-120% for the lowest level) back-calculated accuracies from nominal spanning physiological range concentrations, with 1/x of concentration weight, to compensate for different variance at low concentration, and coefficients of correlation, 0.99, or higher.

Quantification was conducted using MultiQuant 2.1 software (Sciex, Foster City, CA, USA).

22. Byun JA, Lee SH, Jung BH, Choi MH, Moon MH, Chung BC. Analysis of polyamines as carbamoyl derivatives in urine and serum by liquid chromatography-tandem mass spectrometry. Biomedical chromatography : BMC. 2008;22(1):73-80.

23. Magnes C, Fauland A, Gander E, Narath S, Ratzer M, Eisenberg T, et al. Polyamines in biological samples: rapid and robust quantification by solid-phase extraction online-coupled to liquid chromatography-tandem mass spectrometry. Journal of chromatography A. 2014;1331:44-51.
